# Prevalence of Internet gaming disorder and its association with psychiatric comorbidities among a sample of adults in three Arab countries

**DOI:** 10.1186/s43045-023-00280-x

**Published:** 2023-01-24

**Authors:** Tourki Abdulmhsen Almutairi, Khaled Sultan Almutairi, Khaled Mohamed Ragab, Anas Zakarya Nourelden, Ahmed Assar, Sajeda Matar, Hivan Haji Rashid, Mohamed Elsayed, Ahmed Hashem Fathallah, Manfred Spitzer, Carlos Schönfeldt-Lecuona, Collaboration Team, Ebraheem Albazee, Mohamad Klib, Zeina Mohammed Hassan

**Affiliations:** 1 Faculty of Medicine, Jordan University, Amman, Jordan; 2 Faculty of Medicine, Arabian Gulf University, Manama, Bahrain; 3 Faculty of Medicine, Minia University, Minia, Egypt; 4 Faculty of Medicine, Al-Azhar University, Cairo, Egypt; 5 Faculty of Medicine, Menoufia University, Menoufia, Egypt; 6 Faculty of Pharmacy, Jordan University, Amman, Jordan; 7 Faculty of Medicine, University of Aleppo, Aleppo, Syria; 8 Department of Psychiatry and Psychotherapy III, University of Ulm, Leimgrubenweg 12-14, 89075 Ulm, Germany; 9 Department of Psychiatry, School of Medicine and Health Sciences, Carl von Ossietzky University Oldenburg, Oldenburg, Germany

**Keywords:** Gaming disorder, Addiction, Survey, Cross-sectional, Arab population

## Abstract

**Abstract:**

**Background:**

As Internet gaming became publicly available over the past 25 years, Internet gaming disorder emerged as a new diagnostic entity and became established in psychiatric diagnostic systems as a form of addiction. Given the recency of its advent, reliable data on the epidemiology and psychiatric comorbidity of this disorder in specific geographic regions are scarce and dearly needed for appropriate treatment.

**Results:**

A total sample of number = 1332 participants completed the questionnaire. Four-hundred twenty-three of them were gamers; in this cohort, the prevalence of Internet gaming disorder was 6.1%. A strong association between Internet gaming and several psychiatric disorders (attention deficit, hyperactivity, depression, and anxiety) was found.

**Conclusions:**

Internet gaming disorder is frequent in adults from Arab countries. It is associated with psychiatric comorbidities in this current sample; the nature of this association needs to be properly investigated.

## Background

Within the past 25 years, with the advent and widespread use of the Internet, its negative consequences have begun to have an impact on the mental health and well-being of users [1]. Internet addiction has been recognized as a disorder in the *Diagnostic and Statistical Manual of Mental Disorders (DSM-IV)* since 1996, with a range of addictive behaviors enabled by the Internet, such as online gambling, shopping, social media use, and online video gaming [2, 3]. Internet video gaming is also a trigger for various physical and mental disorders [1]. Internet gaming disorder (IGD) has been added in Section III, “Conditions for Further Study” of DSM-5 [4], and has been included in the 11th version of the International Classification of Diseases (ICD-11) under the disorders due to addictive behaviors (6C51 Gaming disorder). Researchers have studied the detrimental impacts of Internet and video game addiction over the past 10 years, particularly concerning children’s physical and mental health. Addictions to the Internet and video games, in particular, have been linked to poor academic performance, the escalation of violence, social exclusion, violent delinquency, antisocial conduct, despair, anxiety, and poor psychological well-being. Researchers have also observed that certain avid gamers display signs of behavioral addiction.

Associations between IGD and other psychiatric comorbidities such as depression, anxiety, obsessive-compulsive disorder, and attention deficit and hyperactivity disorder (ADHD) have already been suggested in the literature [5, 6]. The reported prevalence of IGD varies widely depending on the nation, the age of the sample, and the study technique used to determine the prevalence. It is estimated that 4.7% of the population is affected by this disease on average [7], based on research conducted over the previous two decades. Researchers have shown that younger people and men are more likely to suffer from IGD [8, 9].

However, quantitative information about the emerging condition of IGD among different Arab populations (such as Kuwait, Jordan, and Syria) is lacking. Therefore, we investigated the prevalence of Internet gaming disorder among a sample of gamers from these three Arab countries (Kuwait, Jordan, and Syria) and examined its association with other psychiatric comorbidities such as depression, anxiety, attention deficit, and hyperactivity.

## Methods

In a cross-sectional online survey, the prevalence of IGD and its psychiatric associations among gamers from three Arab countries (Jordan, Kuwait, and Syria) was assessed. The study used social media platforms (Facebook, Twitter, and LinkedIn) to distribute the online questionnaires described below. Any adult from 18 to 35 years from those three countries was eligible to participate in the study; social media platforms were used for data collection through invitations via private messages to participate in the survey. Ethical approval of the study was obtained from the internal review board (IRB) of the Faculty of Medicine, Aleppo University, Syria (number: 8197). The participants were asked to consent that they agree to answer the questionnaire for research purposes on the first page of the online form. At the beginning of the survey, participants were asked if they play Internet games (yes/no). By answering “yes,” they were directly linked to the survey. By answering “no,” they were directed to the questionnaire about psychiatric comorbidities only, which includes three tools to quantify depressiveness, anxiety as well as attention deficit, and hyperactivity (the inventories are described in the “Methods” section). Data were collected in June and July 2021.

The following *demographic variables* were obtained: age, gender, residence, parents’ education, profession, and country of residence.

### Study questionnaires

#### The Internet Gaming Disorder-20 questionnaire (IGD-20)

The questionnaire consists of 20 questions to be answered on a 5-point Likert scale [never [1], rarely [2], sometimes [3], often [4], and very often [5]]. Thus the obtainable score ranges from 20 to 100, with scores above a cutoff point of 71 considered to indicate the presence of IGD. The questionnaire has a Cronbach’s alpha of 0.87 and 0.9, for the original and the Arabic version, respectively [10, 11].

#### The Patient Health Questionnaire (PHQ-9) for the assessment of depression

The questionnaire consists of nine questions to be answered on a 4-point Likert scale [not at all (0), on several days [1], on more than half of the days [2], nearly every day [3]]. Thus, the obtainable score ranges from 0 to 27, with higher scores meaning higher levels of depression. The Cronbach’s alpha for the questionnaire is 0.89 and 0.88, for the English and Arabic versions, respectively [12–14].

#### The Generalized Anxiety Disorder (GAD-7) questionnaire for the assessment of Anxiety

The questionnaire consists of 7 questions to be answered on a 4-point Likert scale [not at all (0), on several days [1] on more than half of the days [2], nearly every day [3]], with a score ranging from 0 to 21, and higher scores indicating higher anxiety levels. The Cronbach’s alpha for the questionnaire is 0.89 for the English and 0.88 for the Arabic version, respectively.

#### The Adult ADHD Self-Report Scale 26 (ASRS-26) (Arabic version)

The questionnaire consists of nine questions assessing attention deficit and nine questions assessing hyperactivity. The score for attention deficit and hyperactivity ranges from 0 to 36 each, with higher scores indicating higher levels of severity. The responses are scored depending on the question as zero or 1. On items, 1–3, 9, 12, 16, and 18 ratings of very often, often, or sometimes are assigned 1 point, while ratings of rarely or never are assigned 0 point. Regarding the remaining 11 items, ratings of very often or often are assigned 1 point (ratings of sometimes, rarely, and never are assigned 0 point) [15, 16].

Participants who reported not playing Internet games were not given the IGD-20 but rather the PHQ-9, GAD-7, and ADHD self-report scale only.

### Sampling and sample size calculation

We used the following equation *n* = z^2^P[1-P]/d^2^ [17], with *z* denoting the statistic corresponding to confidence level, *P* being the prevalence that is expected, and *d* being the precision in correspondence to the effect size. Under the assumptions of a 95% CI, 50% response distribution, and 0.05 margin of error, a sample size of 384 participants was calculated as a minimal sample to represent the population of gamers. We continued to recruit responses (from both gamers and non-gamers) until the minimal sample size of gamers was reached. A team of data collectors (EA, MK, ZMH) was assigned to each country to distribute the online questionnaire on social media platforms, and the responses were collected from the social media platforms. As all three participating countries belong to the Arab region and therefore share the same language, values, and comparable Internet resources, data from the three participating countries were pooled for the analysis.

### Statistical analysis

Descriptive statistics (frequency and percentage) were used to calculate the prevalence of IGD in the study sample. Furthermore, chi-square tests were used to compare the frequency of gaming disorders according to the participants’ demographics (*p*-value was set at 0.05).

To examine the association between IGD and psychiatric comorbidities, the scores of psychiatric disorders were compared between the disordered/non-disordered groups of gamers using the Mann-Whitney tests. Furthermore, the scores of psychiatric disorders were compared between gamers and non-gamers. Further correlation analyses were run between the gaming disorder score and scores in depression, anxiety, attention deficit, and hyperactivity.

## Results

### Demographic characters of the study sample

A total of 1332 participants with a mean age of 24.51 (*SD* = 7.44) years completed the questionnaire. Of the participants, 48.9% were males, 51.3% had a college degree or above, and 91.8% were urban residents. Table 1 shows the demographic characteristics and country of residence of the entire sample of participants.

**Table 1:** Demographic characters of the entire sample (*n* = 1332)

Basic characteristics	***N*** (%)
**Sex**	
Male	651 (48.9%)
Female	681 (51.1%)
**Country**	
Jordan	540 (40.5%)
Syria	447 (33.6%)
Kuwait	345 (25.9%)
**Residency**	
Rural	109 (8.2%)
Urban	1223 (91.8%)
**Education**	
College or above	683 (51.3%)
Below college	649 (48.7%)
**Do you play video games?**	
Yes	423 (31.8%)
No	909 (68.2%)

Playing online Internet games was reported by 423 (31.8%) of all participants, three-quarters of them were males, and 50% had a college degree or above. Table 2 shows the demographic characteristics and country of residence of the gamer’s cohort.

**Table 2:** Demographic characteristics of the subgroup of all gamers

Basic characteristics	***N*** (%)
**Gender**	
Male	315 (74.5%)
Female	108 (25.5%)
**Country**	
Jordan	207 (48.9%)
Syria	114 (27%)
Kuwait	102 (24.1%)
**Residency**	
Rural	36 (8.5%)
Urban	387 (91.5%)
**Education**	
College or above	208 (49.2%)
Below college	215 (50.8%)

### Prevalence of IGD among gamers

**Fig. 1:**
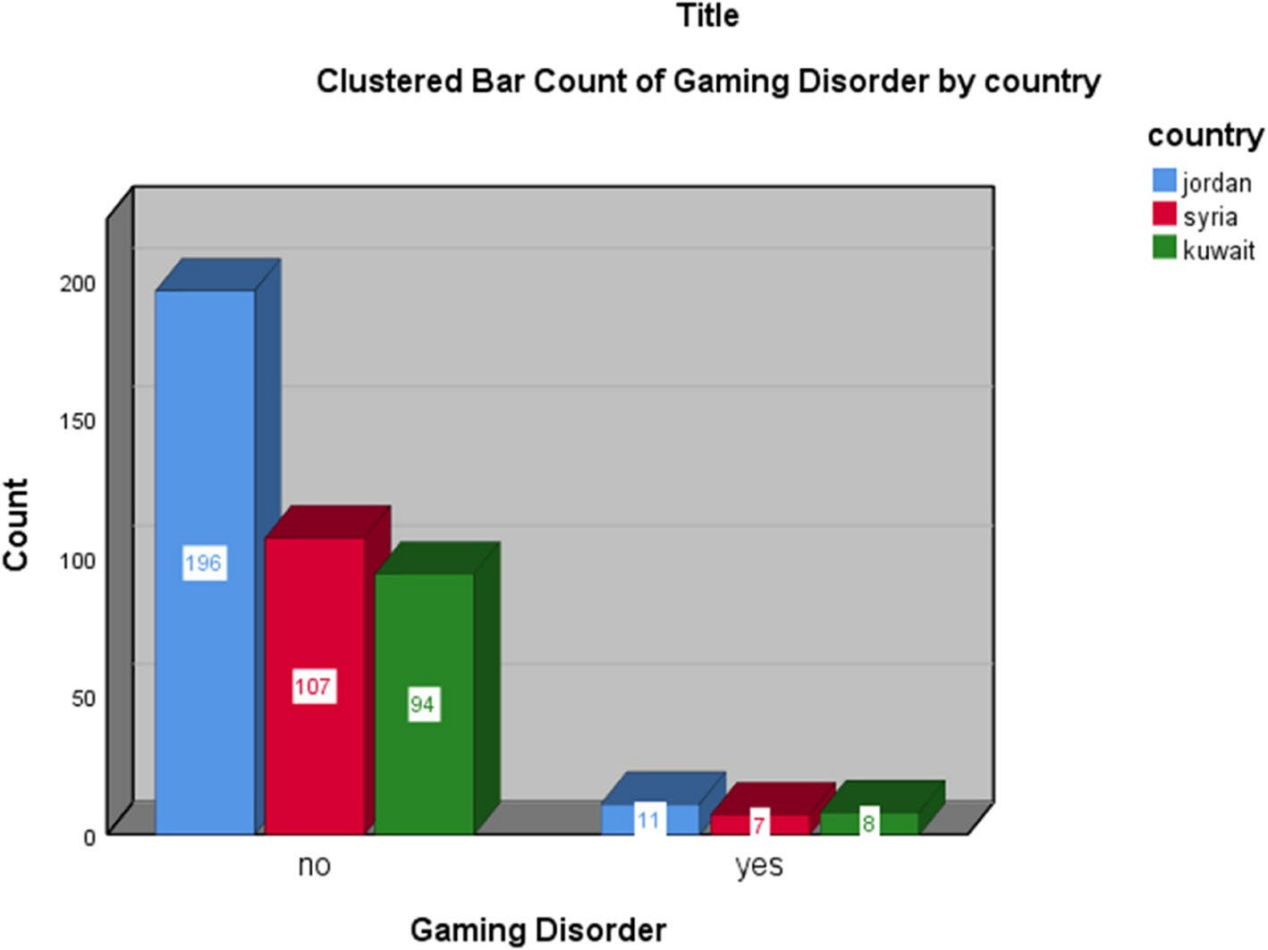
The prevalence of gaming disorder in gamers from Jordan, Syria, and Kuwait

Of the 423 participants that reported playing Internet games, 26 participants were found to be affected by Internet gaming disorder, given the cutoff point of the IGD-20 questionnaire, resulting in a variation in the prevalence of IGD in gamers in the three different countries: 5.3% from Jordan, 6.1% from Syria, and 7.8% from Kuwait (Fig. 1).

### Association between IGD and demographics

The prevalence of IGD was higher in female gamers (10.2%) compared to male gamers (4.8%) (*P* = 0.04). In addition, having a college degree was associated with a lower prevalence of IGD (*P* = 0.04). Table 3 shows the association between IGD and the demographics of gamers.

**Table 3:** Association between IGD and the demographics of gamers

Variables		Disordered		***p***-value
		Yes, ***N*** (%)	No, ***N*** (%)	
**Gender**	**Male**	15 (4.8%)	300 (95.2%)	0.041
	**Female**	11 (10.2%)	97 (89.8%)	
**Residence**	**Rural**	4 (11.1%)	32 (88.9%)	0.17
	**Urban**	22 (5.7%)	356 (94.3%)	
**Education**	**Below college**	18 (8.4%)	197 (91.6%)	0.04
	**College and above**	8 (3.8%)	200 (96.2%)	
**Country**	**Jordan**	11 (5.3%)	196 (94.7%)	0.393
	**Syria**	7 (6.1%)	107 (93.9%)	
	**Kuwait**	8 (7.8%)	94 (92.2%)	

#### Association between Internet gaming/Internet gaming disorder and other psychiatric comorbidities

On average, Internet gamers had a significantly higher score of ADHD than non-gamers (*P* = 0.048) but did not differ significantly from non-gamers regarding depression, anxiety, and hyperactivity. Table 4 shows the association between Internet gaming and psychiatric disorders.

**Table 4:** Association between Internet gaming and psychiatric disorders

Variables		Playing video games		***p***-value
		Yes, ***N*** (%)	No, ***N*** (%)	
**Gaming disorder**	**Yes**	26 (6.1%)	-	-
	**No**	397 (93.9%)	-	
		**Yes, mean (SD)**	**No, mean (SD)**	***p*-value**
**Anxiety**		16 (5.8)	16.06 (5.27)	0.949
**Depression**		21.15 (6.81)	20.27 (6.18)	0.14
**Attention deficit**		24.27 (7.53)	23.07 (6.5)	0.048
**Hyperactivity**		24.4 (7.37)	23.41 (6.61)	0.197

In contrast, participants with IGD had significantly higher levels of ADHD; 92 participants had an inattention subtype, 84 participants had a hyperactivity subtype, and 38 with mixed subtypes. Also, participants with IGD had higher levels of depression and anxiety than those who did not have an IGD (see Table 5). The positive correlations between the IGD-20-questionnaire score and the scores of attention deficit (*r* = 0.234; *p* < .001), hyperactivity (0.235; *p* <. 001), depression (*r* = 0.166; *p* = .003), and anxiety (*r* = 0.1268: *p* = .002) were significant.

**Table 5:** Correlation between gaming disorder and other psychiatric comorbidities

Psychiatric disorder	Internet gaming disorder (IGD)	
	***r***	***p***-value
**Attention deficit**	0.234*	< 0.001
**Hyperactivity**	0.235*	< 0.001
**Depression**	0.166*	0.003
**Anxiety**	0.168*	0.002

*Correlation is significant at the 0.01 level (2-tailed)

## Discussion

This study showed that the prevalence of Internet gaming disorders (IGD) among adult gamers across three Arabic countries is 6.1%, with a clear association between IGD and depression, anxiety, attention deficit, and hyperactivity.

In earlier studies, similar prevalence rates of 6.8% [18] and 9.1% [19], respectively, were reported among adult gamers from other countries. However, prevalence rates as low as 0.2% and 1.3% were reported in other groups of adults [20, 21]. This variability of the prevalence may be attributed to the difference in the demographics and age groups of the included samples.

Some studies that reported comparably higher prevalence rates of Internet gaming disorder — up to 13.1% and 17.7%, were conducted on adolescents and students [22, 23]. Other studies with participants of a younger age range, however, reported prevalence rates comparable to those reported in our study [24, 25].

Urbanicity may be a relevant factor in Internet gaming disorder, as most gamers (more than 90%) were found to live in urban areas [26].

Association of Internet gaming disorder with depression [5, 27], anxiety [5, 27–29], attention deficit [5, 30, 31], and even with hostility and aggression [31, 32], as well as obsessive-compulsive disorder [5, 33], was reported in earlier studies. Thus, our results are in line with these findings and extend them to a different sample. According to the findings of our study, teenagers with IGD had greater levels of depressive symptoms. These discoveries are all noteworthy when seen through the lens of statistics.

The IGD can be either a cause or a result of psychiatric disorders, and there may be mutual exacerbation [34, 35]. By definition, patients with ADHD suffer from decreased impulse control, which may easily lead to engagement in Internet gaming and, further, the development of IGD [36].

Future studies more data on the prevalence of IGD in understudied age groups and populations, given the fact that Internet-based gaming is a pastime performed by hundreds of millions of people worldwide. Moreover, longitudinal studies as well as studies using biological markers of addiction are required to determine the nature of the association between behavioral addictions and other psychiatric disorders. We suggest that schools and families of gamers educate their children by assisting their children in maintaining a schedule that does not interfere with their obligations. They need to be provided with alternatives to their current pursuits, as well as instruction in skills for managing stress and improving self-control. It might be useful to teach kids how to record their gaming sessions since this could help them make fewer rash choices and give them more time for introspection. In addition, we encourage medical professionals to address their patients’ gaming activity, regardless of whether it is a method of coping or the root cause of their patients’ co-occurring disorders of depression and anxiety. The main strong point of this study is that it recruited an understudied population (young adults in three Arab countries). However, the online questionnaire design may have caused response bias, and this represents a limitation of this study. Also, the questionnaire we used could not detect subtypes of ADHD. A further possible confound may be that this study was performed during the COVID-19 pandemic, as several studies demonstrated an increased prevalence of Internet gaming, and possibly of IGD, during the pandemic [37–39].

Finally, identifying the gamers was based on self-report using “yes”/”not” questions, and some gamers may not have identified themselves. Quite possibly, those with already existing problems due to their game addiction. In other words, given the nature of our sampling procedure, we may have missed some more severe cases. Further studies may address this point with different methods of recruitment (and comparisons of the resulting differences regarding the effects).

No attempt was made to explore a correlation between the prevalence of IGD and various types. As we know that certain features of games, such as violence against women, and randomly distributed reinforcement (which is known to have strong addictive effects), further detailed research may result in an addiction likelihood score, which might be used to label Internet games according to the likelihood with which they cause IDG and other psychiatric disorders.

## Conclusions

In the current study sample, the prevalence of IGD in adults from three Arab countries is 6.1%: 5.3% from Jordan, 6.1% from Syria, and 7.8% from Kuwait with a clear association between IGD and other psychiatric disorders. This is in line with the currently existing literature and encourages further research on the detrimental effects of Internet-based computer games on health and society.

## Data Availability

The data are available upon request.

## Abbreviations

ADHD: Attention-deficit/hyperactivity disorder
ASRS: Adult ADHD self-report scale
COVID-19: *Coronavirus SARS-CoV-2*
DSM-IV: *Diagnostic and Statistical Manual of Mental Disorders*
GAD-7: Anxiety assessment tool
IGD: Internet gaming disorder
IRB: Internal review board
IGD-20: Internet gaming disorder-20 questionnaire
ICD-11: International Classification of Diseases
PHQ-9: *Patient Health Questionnaire*
